# EVALUATION OF IDIOPATHIC SCOLIOSIS IN SUBTYPES OF PECTUS EXCAVATUM AND CARINATUM

**DOI:** 10.1590/1413-785220243206e278331

**Published:** 2025-01-10

**Authors:** Davi de Podesta Haje, Guilherme Antunes Barriviera, Marcos Vinícius Santana Silva, Caroline Kaori Maebayashi

**Affiliations:** 1. Hospital de Base do Distrito Federal, Departamento de Ortopedia e Traumatologia, Brasilia, DF, Brazil.; 2. Centro Clinico Orthopectus, Brasilia, DF, Brazil.

**Keywords:** Idiopathic Scoliosis, Pectus Excavatum, Pectus Carinatum, Epidemiology, Escoliose Idiopática, Pectus Excavatum, Pectus Carinatum, Epidemiologia

## Abstract

Objective: Evaluation of epidemiological data on Idiopathic Scoliosis in patients with different pectus subtypes. Methods: A medical record analysis of 418 patients with pectus, associated with idiopathic scoliosis above 10°, with research on: subtypes of pectus (Lateral Pectus Carinatum, Inferior Pectus Carinatum, Superior Pectus Carinatum, Broad Pectus Excavatum, and Localized Pectus Excavatum), and characteristics of the scoliotic curve (Cobb angle, laterality, and location). Results: The mean age was 14.6 years (22° mean Cobb, 52% females and 48% males). The most frequent kind of pectus was Inferior Pectus Carinatum (28%). The left-convex thoracolumbar type was the most frequent. Scoliosis was more severe in thoracic location and females. The main type was LPC (p < 0.05) in those with severe scoliosis. BPE was more present in men (68%), and LPC and SPC in women (p < 0.05). No significant differences were found between the pectus type and the side or location of the scoliosis curve. Conclusion: Depending on the subtype of pectus, the scoliotic curve presents distinct epidemiology and characteristics. *Level of Evidence IV, Case Series – study prognostic.*

## RESUMO

Objetivo: Avaliação dos dados epidemiológicos da Escoliose Idiopática em pacientes com o pectus carinatum e excavatum. Método: Foi realizada uma análise retrospectiva dos prontuários de 418 pacientes com pectus associados a escoliose acima de 10°, sendo pesquisado: subtipos de pectus (carinatum: inferior-PCI, lateral-PCL e superior-PCS; e excavatum: amplo-PEA e localizado-PEL), característica da curva escoliótica (ângulo de Cobb, lateralidade e localização: –torácica, toracolombar e lombar). Resultados: A idade média foi de 14,63 anos na primeira consulta, o ângulo de Coob médio de 22°. O tipo toracolombar sinistro-convexo foi o mais frequente. As escolioses torácicas foram mais severas que as toracolombares e lombares, e tiveram o lado direito como mais frequente. Nas escolioses mais severas o principal tipo foi o PCL(p<0,05). O PEA foi mais presente em homens (68%), e o PCL e PCS em mulheres (p<0,05). Não foram encontradas diferenças significativas entre o tipo de pectus e o lado ou local da curva da escoliose. Conclusões: A depender do subtipo de pectus, a curva escoliótica apresenta epidemiologia e características distintas. *Nível de Evidência IV, Série de Casos – Estudo Prognóstico* .

## INTRODUCTION

 The set of anterior chest wall deformities is universally defined as pectus. ^1^ It has an incidence of 0.2% to 3% of the population with a report of 1:300/400 live births and a ratio of 3/5:1 in men. ^2^ ^-^ ^4^

 A chest wall protrusion deformity is defined as pectus carinatum, while pectus excavatum is the depression deformity. In addition, they can be subdivided regarding the location of the apex of the deformity, classifying them as Superior (SPC) / Inferior (IPC) / Lateral (LPC) for pectus carinatum, and as Localized (LPE) / Broad (BPE) for pectus excavatum. ^5^

 Idiopathic scoliosis is a three-dimensional deformity of the spine defined by a Cobb angle greater than 10° without a defined cause. It has an incidence of 2–3% in the general population, being more common in females from adolescence onwards. It is subdivided according to the age it appears, into infantile (0–3 years old), juvenile (4–10 years old), and adolescent (> 10 years old). ^6^ ^,^ ^7^

 After the first description of the idiopathic scoliosis association with pectus by Walter in 1989, other studies reported the incidence of these pathologies together. ^4^ ^,^ ^8^ ^,^ ^9^ These studies showed increased incidence compared to the normal population (17.61% – 28.6%). Hong et al. reported a higher incidence of females having both pectus and scoliosis. Zhong et al. reported a higher incidence of right-convex curves in those with pectus, and Wang et al. reported that the higher the Haller, the greater the severity of scoliosis. 

There are no studies evaluating the incidence and characteristics of scoliotic curves in the pectus subtypes.

Thus, in view of the importance of knowledge for these pathologies proper monitoring, this study aims to analyze the scoliosis epidemiology in the pectus subtypes.

## MATERIAL AND METHODS

 Data was collected for patients diagnosed with Pectus Excavatum and Carinatum and Idiopathic Scoliosis treated at two pectus centers from 1995–2023 by two pediatric orthopedists references in the non-surgical treatment of pectus. *This study was performed in line with the principles of the Declaration of Helsinki. Approval was granted by the Ethics Committee* under CAAE number 70798923.3.1001.8153. A written informed consent was obtained from the parents. For 7,101 medical records of patients with pectus, 2,225 (31%) had a record of scoliosis, 418 of which had complete information necessary for the study. Data on age of the first appointment, gender, type of pectus, and characteristic of the scoliotic curve were collected from the medical record. 

 The pectus was classified as described by Haje: ^5^ as Lateral Pectus Carinatum (LPC), Inferior Pectus Carinatum (IPC), Superior Pectus Carinatum (SPC), Broad Pectus Excavatum (BPE), and Localized Pectus Excavatum (LPE). 

The following characteristics of scoliosis have been researched: (a) the Cobb angle of the largest curve (scoliosis severity was defined as: Mild up to 20°, Moderate from 20–50°, and Severe above 50°); (b) laterality of convexity; and (c) affected segment: Thoracic (T); Thoracolumbar (TL), and Lumbar (L). The type of scoliosis was defined by laterality (right/left) and affected segment.

Statistical analysis:

The chi-squared test or Fisher’s exact test were used to compare categorical variables. To compare age among pectus groups, the Analysis of Variance (ANOVA) method with Bonferroni correction was applied. For statistical significance, p ≤ 0.05 was adopted in all analyses.

The following variables were statistically related:

- Gender x Severity of scoliosis
- Curve severity x Location of scoliosis
- Location of scoliosis x Side of scoliosis
- Type of pectus x Gender
- Type of pectus x Laterality of scoliosis
- Type of pectus x Type of scoliosis
- Type of pectus x Location of scoliosis
- Type of pectus x Scoliosis severity
- Type of scoliosis x Age at first appointment

## RESULTS

The study presented a group of 215 (52%) women and 203 (48%) men. The mean age at the first evaluation appointment was 14.63 years old, ranging from 2.06 to 50 years old. The mean Cobb Angle was 22° (Moderate), ranging from 10° to 90°. The most frequent curve was Thoracolumbar, corresponding to 306 cases (73.2%), and the most frequent segment was T12-L4 (11%). In addition, 50.5% of the cases were right-convex scoliosis and 49.5% were left-convex scoliosis.

Within the analysis of the scoliosis, it was found a significant difference in relation to:

 a) Scoliosis severity and gender (p < 0.001*; χ ^2^ = 26.22), more common in females.The male gender had a significantly higher incidence in the Mild scoliosis type (68.5% > 45.1%), while the percentage of females was significantly higher in the Moderate type (49.8% > 25.6%). 

b) Scoliosis severity and location (p = 0.002), the most severe curves being Thoracic. The percentage of Mild scoliosis is significantly higher in the Lumbar location, when compared to T (75% > 38.1%); and the percentage of Moderate scoliosis is significantly higher in T compared to L (53.6% > 25%).

 c) Scoliosis location and the side of the curve (p < 0.001*; χ ^2^ = 36.20), with the most prevalent being Thoracolumbar scoliosis with left curve, present in 41.9% of the cases. The percentage right curves was significantly higher in Thoracic scoliosis in relation to Lumbar and Thoracolumbar (79.8% > 46.4% and 42.8%). By symmetry, the percentage of left curves is significantly higher in Locations L and TL in relation to T (57.28% and 53.6% > 20.2%). 

About the pectus subtypes, the prevalence was: IPC (118, 28.2%), LPC (110, 26.3%), SPC (19, 4.54%), BPE (79, 18.8%), and LPE (92, 22%).

 Regarding the type of pectus and gender, there is a significant difference (p < 0.001*; χ ^2^ = 25.62). The percentage of males is significantly higher in Broad Pectus Excavatum (BPE) compared to all other pectus (69.6%; p ≤ 0.05 in all comparisons). The percentage of males is significantly higher in Inferior Pectus Carinatum (IPC) than in Lateral Pectus Carinatum (LPC) (50.8% > 35.5%, p ≤ 0.05). In a complementary way, for the female gender, BPE has lower incidence than the other pectus (30.4%); the percentage of females is significantly higher in LPC compared to IPC (64.5% > 49.1%, p ≤ 0.05). 

 No statistical differences were observed between the type of Pectus and laterality of the scoliosis (p = 0.59; χ ^2^ = 2.81); the type of Pectus and type of scoliosis (e.g., right thoracolumbar) (p = 0.12; χ ^2^ ); the type of Pectus and scoliosis location (p = 0.31; χ ^2^ = 9.37); the type of scoliosis and age at first appointment (p = 0.13). 

 There is no general difference (p = 0.11; χ ^2^ = 12.83) comparing pectus with scoliosis severity, however, fixing the sample in the subtypes, there is a difference. Among the Severe ones, there is a difference between the percentages (LPC 47.8% > SPC 4.3%, BPE 13%, and LPE 13%) (p = 0.01*; χ ^2^ = 12.87). Among the Moderate cases, there is difference between the percentages (LPC 28.3%, IPC 31.4%, BPE 18.2%, and LPE 18.2% > SPC 3.8%) (p < 0.001*; χ ^2^ = 38.05). Among the Mild ones, there is difference between the percentages (LPC 26.8%, IPC 20.8%, BPE 19.9%, and LPE 25.4% > SPC 5.1%) (p < 0.001*; χ ^2^ = 39.81). 

## DISCUSSION

 The idea of the correlation between Pectus and Scoliosis arises from clinical observations of patients with chest deformities associated with syndromes such as Marfan syndrome. ^10^ Subsequently, the correlation was theorized based on the anatomical precept of the thorax — anterior section with the sternum and posterior section with the spine — and a joint treatment was even proposed for the two deformities. ^11^ In addition, studies have shown the genetic correlation between the two pathologies. ^12^ Gurnett et al. demonstrated the correlation of both pathologies with chromosome 18q. 

 Pectus, seen as an isolated entity, is considered the most common congenital deformity of the chest wall. It is more common in men in proportions of 4:1 in the literature, ^2^ ^,^ ^13^ but it is characterized differently in the specific pectus/scoliosis population. ^4^ In our study, in agreement with the findings of Hong et al., a higher prevalence of females was observed. This difference seen in the pectus/scoliosis population may be influenced by scoliosis. In our study, however, in comparison to the Pectus subtypes, it was found a higher prevalence of males in Broad Pectus Excavatum (BPE). 

 The Pectus is classified according to the Carinatum or Excavatum deformity, however, it can also be classified by the deformity position according to Haje’s classification. ^5^ Although most of the literature reports that the pectus excavatum is more frequent, our study was in line with other previous studies that showed the highest prevalence of pectus carinatum in the pectus/scoliosis population. ^14^ ^,^ ^15^ According to a previous study by Haje, the prevalence of the type of pectus remained, with the Inferior Pectus Carinatum (IPC) being more prevalent, followed by the Lateral Pectus Carinatum (LPC), and the Localized Pectus Excavatum (LPE) in third place. It was seen, however, that the proportion of LPC in those with pectus and scoliosis was much higher in our study than that reported by Haje in a study reporting a global sample of pectus. Furthermore, the proportions of excavatum in the pectus/scoliosis population increased in relation to the isolated pectus population. In the pectus isolated group Carinatum 78% x Excavatum 22%, ^1^ while in the Pectus/scoliosis population Carinatum 59% x Excavatum 41%) 

 In the analysis of scoliosis, especially in adolescents, a higher percentage of the female gender is found in proportions of 1.5/3:1. When evaluating the Cobb angle severity, the numbers go to 7.2:1. ^16^ In our study, we found that Mild scoliosis were more prevalent in males, and Moderate and Severe conditions more prevalent in the females. The scoliosis location and the side of the curve are, however, distinct from the epidemiology of scoliosis. The literature treats the incidence of Thoracic scoliosis with right curve as the most common, ^16^ ^,^ ^17^ while our study found Thoracolumbar scoliosis with left curve as the most prevalent. 

Comparison of scoliosis with pectus subtypes did not show significant differences between the side of the curve, the scoliosis location, and the scoliosis type. We found a significant correlation between the LPC type associated with Severe scoliosis, when compared to the other subtypes. Our theory is that Severe scoliosis cases present vertebral body rotation, causing the rotation of the entire hemithorax to be secondary to the rotation of the ribs inserted in the affected vertebrae, which may generate asymmetries in only one hemithorax, or a compatible LPC condition.

Patients with pectus associated with scoliosis had a distinct epidemiology from those with isolated pectus or isolated scoliosis, with more frequent left-convex thoracolumbar curves. Scoliosis in pectus patients was more severe in females, in those with right-convex thoracic curves, and in the LPC subtype.

## CONCLUSION

Depending on the pectus subtype, the scoliotic curve has different epidemiology and characteristics.
